# Oral Mucosa as a Potential Site for Diagnosis and Treatment of Allergic and Autoimmune Diseases

**DOI:** 10.3390/foods10050970

**Published:** 2021-04-28

**Authors:** Cristina Gomez-Casado, Javier Sanchez-Solares, Elena Izquierdo, Araceli Díaz-Perales, Domingo Barber, María M. Escribese

**Affiliations:** 1 Institute of Applied Molecular Medicine, Department of Basic Medical Sciences, Faculty of Medicine, San Pablo CEU University, 28003 Madrid, Spain; j.sanchez127@usp.ceu.es (J.S.-S.); elena.izquierdoalvarez@ceu.es (E.I.); domingo.barberhernandez@ceu.es (D.B.); mariamarta.escribesealonso@ceu.es (M.M.E.); 2 Center of Plant Biotechnology and Genomics, Technical University of Madrid, 28040 Madrid, Spain; araceli.diaz@upm.es

**Keywords:** oral mucosa, food allergy, diagnosis, treatment, desensitization, celiac disease, inflammatory disease, autoimmune disease, systemic disease

## Abstract

Most prevalent food allergies during early childhood are caused by foods with a high allergenic protein content, such as milk, egg, nuts, or fish. In older subjects, some respiratory allergies progressively lead to food-induced allergic reactions, which can be severe, such as urticaria or asthma. Oral mucosa remodeling has been recently proven to be a feature of severe allergic phenotypes and autoimmune diseases. This remodeling process includes epithelial barrier disruption and the release of inflammatory signals. Although little is known about the immune processes taking place in the oral mucosa, there are a few reports describing the oral mucosa-associated immune system. In this review, we will provide an overview of the recent knowledge about the role of the oral mucosa in food-induced allergic reactions, as well as in severe respiratory allergies or food-induced autoimmune diseases, such as celiac disease.

## 1. Introduction

The oral mucosa or mucosal lining consists of primarily non-keratinized stratified squamous epithelia and a highly vascularized connective tissue called the lamina propria, which underlies the epithelia [1]. The oral mucosa is an immunocompetent site, whose function is primarily tolerogenic [1,2,3]. It lines the inside of the mouth and therefore acts as a physical barrier; nevertheless, it also contains immune cells that help maintain mucosal homeostasis [1]. Therefore, an intact and fully functional oral mucosa is crucial to prevent immune reactions to innocuous environmental antigens [4].

In several allergic and autoimmune diseases, the oral mucosa has been proven to undergo a remodeling process. This remodeling is characterized by a leaky epithelial barrier, a fibrotic lamina propria, the release of inflammatory mediators, and the recruitment of immune infiltrate [5,6,7].

The aim of this review is to examine the growing knowledge on the characteristics of oral mucosal remodeling in allergic (food allergy, respiratory allergy) or non-allergic (i.e., celiac disease, a food-induced autoimmune) diseases. Understanding oral mucosa remodeling features, in addition to these being a potential tool for diagnosis, will provide a rationale to develop oral therapeutic strategies that may help prevent or treat these pathologies.

## 2. Oral Mucosa

### 2.1. Structure of the Oral Mucosa

Despite the continuous exposition of the oral mucosa to external stimuli, pathological events are not so frequently seen. This is due to the anatomical and physiological features of the oral mucosa and possibly to the limited exposition time to external stimuli. The oral mucosa comprises three layers: the epithelium, the basement membrane, and the lamina propria, and contains active networks of extracellular matrix, different cell types, and neuro-vascular systems [8].

Interestingly, oral mucosa structure varies along its location within the oral cavity, but three main types of mucosa can be recognized based on their morphology and specific pattern of differentiation: keratinized stratified squamous epithelium- masticatory mucosa, which covers the hard palate and gingiva; non-keratinized stratified squamous epithelium- lining mucosa, on the underside of the tongue, the inside of the lips, cheeks, the floor of the mouth, and the alveolar ridge; and the specialized mucosa of the dorsal surface of the tongue [8,9,10].

The oral epithelium is the superficial layer that separates the environment from underlying tissues. It is a stratified squamous epithelium consisting of cells tightly attached to each other and arranged in layers. It possesses structural properties such as stratification and cornification of the keratinocytes and specific cell-to-cell interactions to maintain its barrier function [8,9,10]. The keratinized type contains four layers of cells: the basal layer, spinous layer, granular layer, and the superficial layer (keratinized layer). Keratinocytes are born and proliferate in the basal layer and undergo terminal differentiation as they migrate to the surface where they die. Thus, the outermost cell layer is dead cells. Conversely, the surface cells of non-keratinized epithelia are living cells without keratin. Moreover, the non-keratinized oral epithelium has no granular layer [8,9,10].

The integrity of the epithelial barrier is a key factor to avoid uncontrolled antigen penetration. Oral epithelium integrity is maintained through highly specific junctional complexes between epithelial cells. Three types of epithelial cell junctions have been described: tight junctions (TJs), gap junctions (GJs), and anchoring junctions [10,11,12,13]. Tight junctions form the closest cell–cell interactions in the apical area of oral epithelial cells, working as a restrictive gate for the passage of water, electrolytes, and other small molecules. They consist of a number of transmembrane proteins, including occludin, claudin, and immunoglobulin-like surface, as well as cytoplasmic molecules such as zonula occludens (ZO) [10,11]. Gap junctions are composed of hemichannels called connexons which are regulated by several factors, including pH, calcium concentration and posttranslational modifications. Thus, they provide direct communication between adjacent cells and the exchange of small molecules and ions. Anchoring junctions are classified as adherens junctions (AJs), desmosomes, and hemi-desmosomes [12]. Adherens juntions are protein complexes situated below TJs that strongly hold cells. Whereas, AJs are composed by cadherins, such as E-cadherin, that connect to the actin cytoskeleton by catenins; desmosomes link two cells together by the intermediate filament cytoskeleton, becoming the adhesive bonds that give mechanical strength to tissues. Moreover, desmosomes act as surface receptors that mediate cellular differentiation and proliferation. Adhesion between the epithelium and connective tissue is provided by hemidesmosomes (half of a desmosome), which link the intermediate filament network of epithelial cells to the underlying basement membrane [11,12]. Since the structure and functions of all of the abovementioned cell–cell junctions are key to preserve epithelial barrier integrity, their disruption has been linked to infections, autoimmune diseases, allergies, and cancer disorders [11,14,15,16,17,18,19]. Epithelial cells in the mucosa, which were initially thought to be just inert barriers, are now known to play a key role in the immune-protective system of the mucosa [2,20,21,22]. Epithelial cells can react to external stimuli by synthesizing cytokines, adhesion molecules, growth factors, chemokines, and matrix metalloproteases [10]. Likewise, inflammatory factors, such as IFNγ and TNFα, have been reported to disrupt epithelial integrity through downregulation of TJ proteins, increasing epithelial permeability [23,24,25,26,27,28]. Generation of epithelium-derived cytokines, such as thymic stromal lymphopoietin (TSLP), IL-25, and IL-33, leads to Th2 immunity [29].

The basement membrane or basal lamina is a thin layer composed of collagen and laminin situated between the epithelium and lamina propria. It appears as a structureless band and includes glycogen, muco-substances, glycolipids, and phospholipids [8].

The lamina propria is the underlying connective tissue and consist of networks of fibers arranged in groups and a ground substance composed of water, glycoproteins, and proteoglycans, and serum-derived proteins. Moreover, it contains a variety of cells, blood vessels, and neural elements [8,30]. Fibroblasts are the principal cell of the lamina propria, whose main function is maintaining tissue integrity by secreting fibers and ground substance [8,30].

### 2.2. Oral Mucosa-Associated Immune System

The oral mucosa is an immunocompetent site [3,31] that displays lymphoid tissue foci where antigen-presenting cells (APCs) and lymphocytes co-localized in the lamina propria [1,3,31]. In homeostatic conditions, the lamina propria harbors small numbers of lymphocytes and dendritic cells (DC), which can become the dominant cell type in chronic conditions. Whereas, in response to infection (acute condition), the recruitment of polymorphonuclear leukocytes (mainly neutrophils), monocytes, and macrophages represents an important component of the innate immune response [1]. Macrophages of the lamina propria are scavengers of damaged tissue and foreign material. Additionally, they can present the processed ingested material to T cells and produce cytokines and chemokines that stimulate fibroblast proliferation and collagen production necessary for repair [1,8]. Primarily located adjacent to the basement membrane of endothelial cells, mast cells containing granules with histamine, heparin, and cytokines are found. Their migration is influenced by the synthesis of mast cell growth factor by endothelial cells and keratinocytes [1,8].

In response to any insult, the abundant vasculature ensures the recruitment of inflammatory cells. In turn, the oral epithelium responds to irritation by an increase in cell proliferation and hyper-keratinization. Moreover, the oral epithelium reacts to microorganisms by secreting antimicrobial peptides, antimicrobial proteins (lytic enzymes), chemokines, cytokines, and neuropeptides. These molecules are also produced by polymorphonuclear cells, myeloid cells, keratinocytes, and fibroblasts. Similarly, capillaries of the lamina propria express adhesion molecules which facilitate the trafficking of leukocytes from the blood [1,8,10].

The tolerogenic properties of the oral mucosa seem to rely on their specific types of APC, which are differently distributed along mucosal sites [1,3,32]. In mice, four subtypes of oral CD11c+ DCs have been identified depending on the expression of CD103, Ep-CAM (epithelial cell adhesion molecule), and langerin+ (Ln+) surface markers: interstitial DCs (iDCs), Langerhans cells (Ln+Ep−CAM+), Ln+ iDCs (CD11c+Ep−CAM+), CD103+ iDCs (CD11c+) [32]. The CD103+ DCs may also express CD207 and are the main migratory subtype able to cross-present viral and self-antigens, critical for the initiation of CD8+ T cell responses [33]. Plasmacytoid DCs (B220+120G8+) have been also found on the sublingual mucosa area [34]. Notably, CD11c−CD11b+ APCs (presumably macrophages) can be detected in low numbers in the buccal mucosa but predominate in the mucosa of the sublingual area [34].

In human oral mucosa, Langerhans cells (Ln+CD1a+) represent the predominating APC population, but myeloid DCs have also been detected [35]. They constitutively express FcεRI and display upregulated levels of co-inhibitory molecules (B7-H1 and B7-H3), but a decrease in the co-stimulatory molecule expression CD86. Moreover, oral Langerhans cells express MHC class II and the CD83 maturation marker. All these features contribute to oral Langerhans cells’ induction of T regulatory (Treg) cells and the secretion of IL-10 and TGFβ, which explain their role as tolerogenic cells [35]. Oral and epidermal Langerhans cells, which are constantly exposed to microbial stimuli, express similar levels of the CD83 maturation marker, suggesting a state of maturation associated with tolerance induction [35]. Analysis of the gingiva identified Ln+DCs in the mucosal epithelium and CD209+ DCs in the lamina propria, which are considered the equivalent of dermal DCs. Contrary to the findings in mice, plasmacytoid DCs characterized by CD123+ are rarely detected in healthy human oral mucosa, although their number increases upon inflammation [36,37].

Within the gingiva, T cells, B cells, and innate lymphoid cells (ILCs) can be detected in healthy conditions [38]. ILCs present in the oral mucosa may help maintain barrier function and protect against pathogenic infections [39]. However, the homeostatic role of B cells and ILCs in oral immune responses to commensals and dietary antigens is still unclear. On the contrary, many studies have described the presence and role of different T cell populations. At murine and human gingiva mucosa, resident memory T cells (mainly CD4+), IL-17 secreting cells (both Th17 and T cell receptor (TCR) γδ T cells), and resident Foxp3+ regulatory T cells (Tregs) have been detected [38,40,41]. Oral T memory cells provide defense against pathogens, while Tregs participate in oral tolerance [1]. Regarding the origin of gingival Tregs, some studies indicate CCR4/CCL22-controlled Treg trafficking [42,43], and a recent investigation revealed that oral murine CD103−CD11b+ DCs possessed the necessary properties to induce Foxp3+ Treg cells [44]. They could show that oral murine CD103−CD11b+ DCs transport sublingual antigens to submandibular lymph nodes and induce antigen-specific Treg cells.

## 3. Oral Mucosa in Food Allergy

The interaction between epithelial and immune cells at the mucosal linings has been proven to be critical in the onset and maintenance of allergic inflammation [5]. Therefore, an intact and fully functional mucosal barrier is considered crucial in the maintenance of mucosal homeostasis, as it protects the host immune system from the exposure to allergenic molecules and noxious environmental triggers [21]. The oral mucosa is the first immune tissue that encounters allergens upon ingestion of food. Oral exposure to allergens is complex in terms of immunological effects. In the relatively short time that food proteins are in the mouth, they should be bio-accessible. Therefore, the bio-accessibility of allergens at this stage may be a determinant for sensitization [45]. The continuous exposure of the oral mucosa to environmental triggers (antigens, allergens, or contaminants) induces a remodeling process in the oral epithelial barrier. This remodeling is maintained over time. An impaired oral mucosal barrier can therefore facilitate allergen access. Additionally, the damaged epithelium secretes the alarmins IL-25, IL-33, and TSLP. This triggers a local inflammatory immune response characterized by increased expression of pro-inflammatory cytokines and higher numbers of immune cells being recruited to the oral mucosa [5]. The chronic exposure to the allergen and its access to the mucosa-associated immune system together with the sustained local inflammation is reflected systemically, e.g., by increased IL-33 plasma levels [5,46]. This results in a positive feedback loop promoting further remodeling and inflammatory events in an otherwise pro-tolerogenic environment.

Food allergy is defined as “an adverse health effect resulting from a specific immune response that occurs reproducibly on exposure to a particular food” [47] that is causing a growing clinical problem. IgE-mediated reactions usually occur within two hours (they can even occur after few minutes) of ingestion of food and affect the skin, gastrointestinal tract, respiratory tract, and, less frequently, the cardiovascular system. In the most severe cases of anaphylaxis, multiple organ systems are involved and may include cardiovascular collapse.

It is currently not understood why some people develop allergic sensitization to foods while most people are immunologically tolerant, but the evidence suggests that environmental factors are important. The increased exposure to biological and chemical air pollutants such as protease enzymes, tobacco, or particulate matter (the so called exposome) has been proven responsible for disrupting the integrity of the epithelial barrier by degrading the intercellular junctions and triggering epithelial alarmin release [48]. The epithelial disruption leads to Th2 immune responses responsible for allergy development [49,50,51]. The “barrier regulation” hypothesis [52] postulates that allergic sensitization begins with the damage of the epithelial barrier [48,53]. Individuals with food allergies have their barrier permeability increased [54,55,56]. Thus, barrier impairment may itself lead to a predisposition toward atopy [57].

Moreover, the route of exposure is another crucial factor in food allergy. Food allergens brought in contact by non-oral routes contribute to allergic sensitization. In fact, epidemiological studies in humans indicate that non-oral contact with food allergens is correlated with the risk of a child developing food allergy [58,59,60]. In addition, symptomatic food allergy is often observed when a child eats the allergenic food for the first time, which is consistent with a previous sensitization phenomenon by non-oral routes [31]. Although there is growing evidence to support a disrupted and inflamed skin barrier as being responsible for the development of food sensitization [61], results from a large randomized controlled trial for the prevention of food allergy were negative [62]. The BEEP (Barrier Enhancement for Eczema Prevention) evaluated whether applying petrolatum-based oils or moisturizers from the first few weeks of life could prevent atopic dermatitis and food allergy and, on the contrary, found an increased rate of infections and a trend toward increased food allergy in the intervention group [62,63], in accordance with the PreventADALL trial on atopic dermatitis [63]. The role of epicutaneous sensitization in the development of food allergy has been extensively reviewed recently [57]. Regarding molecular mechanisms, allergic sensitization is thought to require the activity of T cells that express Th2 cytokines, such as IL-4 and IL-13. However, the exact nature of the T cell support required for the allergen-specific B cells to turn into the IgE-producing plasma cells in humans has not been described yet. Similarly, it is not clear to what extent the change to IgE class occurs in various tissues of the body. In addition, other cell types, such as tissue-resident mast cells, secrete IL-4, IL-13, and other cytokines that can influence the differentiation of B cells [64]. High titers of allergen specific IgE antibodies of high affinity are often detected in patients with symptomatic allergy. These antibodies bind to FcεRI in tissue-resident mast cells and circulating basophils, where they are involved in early or immediate hypersensitivity responses when interacting with allergens. It has also been reported that allergen-specific IgE may contribute to the pathogenesis of allergies by facilitating antigen presentation and epitope spread by means of the uptake of antigen–IgE complexes by the low-affinity IgE receptor, CD23, present on DCs, B lymphocytes, and other APCs [65,66,67,68]. IgE can also help transport the antigen from the lumen via CD23 receptors on the surface of epithelial cells, as it has been shown in the human gut, in cultures of human respiratory epithelial cells [69], and in a mouse model of allergy [70].

In the case of profilin sensitization, the oral mucosa has been proven to be altered and is associated with disease progression [71,72,73,74,75,76]. Profilin is a pollen aeroallergen that normally plays a limited role as a food allergen because it is easily degraded by proteases and acidic conditions. However, it can sensitize some pollen allergic individuals in areas of high allergen exposure. In the study by Rosace et al. [5], grass pollen allergic patients from overexposed areas in Spain were subjected to an oral challenge with profilin. The observed reactions ranged from local reactions such as oral allergy syndrome (OAS), angioedema, and oral pruritus, to severe systemic reactions such as urticaria and asthma. The patients that were allergic to profilin presented a progressive oral mucosal remodeling, characterized by decreased expression of TJ (claudin-1 and occluding) and AJ (E-cadherin) proteins, which led to a leaky epithelial barrier, increased angiogenesis and acanthosis, and augmented collagen deposition in the lamina propria. These processes have been previously associated with mucosal remodeling [77,78,79] and are comparable with those described from patients with other inflammatory pathologies [80,81]. In addition, an increased IL-33 expression was also observed in the severe allergic patients, i.e., those orally sensitized to profilin with a clinical history of severe allergic reactions. The epithelial damage might be associated with the IL-33–dependent ILC2 population located in the mucosal epithelium [82,83,84]. As profilin is present in all vegetables, it would contribute to sustaining the inflammatory allergic response, thereby causing allergic reactions to food [85]. This suggests that oral epithelial remodeling could be a key process for the acquisition of a severe allergic phenotype in patients with profilin-mediated food allergy.

In addition, the bio-accessibility of allergens at the oral cavity may be key to induce oral remodeling and allergic reactions. Allergen bio-accessibility may be modulated by the composition, volume, and pH of saliva [45]. Koppelman et al. [45] investigated the release of peanut allergens from lightly roasted peanut flour in the saliva in different conditions. The allergens Ara h2 and Ara h6, which are the most potent peanut allergens [86,87,88], were rapidly released from the food matrix, while Ara h1 and Ara h3 were poorly released. Therefore, Ara h2 and Ara h6 may be the first peanut allergens that individuals are exposed to upon ingesting lightly roasted peanut flour and may trigger immune responses in the oral mucosa. It remains to be determined whether this is also the case for other peanut-containing foods. Their early release provides them with the unique opportunity to interact with the oral mucosal immune system, which, in the case of accidental ingestion of hidden peanut allergens, can provoke life-threatening anaphylactic reactions in peanut-allergic patients [89,90,91].

## 4. Oral Mucosa in Other Allergies and Inflammatory Pathologies

### 4.1. Oral Mucosa in Respiratory Allergies with No Associated Food Allergy

As it has been previously described, grass pollen allergic patients that suffer profilin-mediated food allergy experience severe reactions and undergo oral mucosa remodeling [5]. However, we have observed that respiratory allergy with no concomitant food allergy can per se cause oral epithelial disruption. Respiratory allergic patients presenting severe allergic reactions such as asthma or urticaria in the absence of food allergy also presented a disrupted oral epithelium and highly fibrotic lamina propria when compared to healthy controls. This was observed in two patient cohorts with respiratory allergy to either olive pollen or house dust mite (HDM). This means that oral mucosa remodeling occurs regardless of the allergen trigger [6]. However, in contrast to profilin allergic patients, olive pollen or HDM allergic subjects showed no augmented angiogenesis or higher immune cell numbers, except for the Treg population found to be increased in the lamina propria of HDM allergic patients. Moreover, eosinophils and neutrophils were hardly present in the oral mucosa of any study subjects [6]. Based on these results, it seems that the structural changes of the oral mucosa take place independently of the recruitment of inflammatory infiltrate, which is presumably directed to the airway mucosa. Moreover, respiratory allergic patients presented increased IL-33 and IL-25 plasma levels (unpublished data). Under inflammatory conditions, epithelial cells can release IL-33. This alarmin disrupts the epithelial barrier and activates ILC2s. ILC2s and Th2 cells and type 2 cytokines have TJ-disruptive effects and induce IgE production [46]. Based on these data, we hypothesize that systemic changes may reflect the leaky mucosal barrier found in the patients of the study by Sanchez-Solares et al. [6]. An impaired epithelial barrier could be responsible for the progression of respiratory to food allergy when an allergen comes into direct contact with the leaky oral mucosa [5]. As a matter of fact, many food allergies (PR10, lipid transfer proteins, profilins) have been associated with previous respiratory sensitizations [92].

### 4.2. Oral Contact Mucosal Allergies

There are several oral contact pathologies that are not frequent in the oral mucosa due to its relative resistance to irritant or allergenic agents but cause immune reactions with broad symptoms. Among them, allergic contact stomatitis is caused by oral flavorings, preservatives, and dental materials [93,94] which causes erythema, edema, erosions, and ulcerations of the entire oral mucosa. Similarly, allergic contact cheilitis is a superficial inflammation of the lip that is usually caused by cosmetic products [93,95]. Geographic tongue or benign migratory glossitis is a benign, usually asymptomatic disorder, which appears as depapillated areas of the tongue with a yellowish or grayish white color [96]. Allergy has been suggested as a major etiologic factor, and nickel sulfate is the most frequent hapten trigger [97]. Similar to nickel, titanium (Ti) in micro or nanoparticles is widely used in dental implants, foods, and cosmetics [98,99]. Ti nanoparticles rapidly interact with the mucosa and may impact on the physiological homeostasis of buccal and sublingual cells in the oral cavity [100,101,102]. Some data indicate that the exposure of gingival cells to Ti resulted in increased expression of Toll-like receptor 4 and intercellular adhesion molecule 1 [103]. Moreover, Ti has been reported to activate macrophages [104,105]. Contact allergens such as dental material or allergenic foods have been identified as triggers of burning mouth syndrome (BMS), a complex disorder characterized by a burning sensation in the oral mucosa without any visible lesions. [93,106,107,108].

### 4.3. Oral Mucosa in Celiac Disease (CD)

Although considered a primary gastrointestinal disease, CD is now known to have widespread systemic manifestations [109]. Many authors have described a wide variety of disorders in the oral cavity of CD patients, some of them being key in the diagnostic of atypical forms of the disease [110,111,112]. As previously detected in allergic patients, we have recently observed a significant decrease in the expression of oral epithelial junctional proteins in CD patients, even after gluten avoidance for at least one year. This indicates that the integrity of the oral epithelial barrier is also compromised in these patients [7]. As observed in olive pollen and HDM respiratory allergy [6], the oral mucosa of CD patients was also impaired without major immune cell recruitment, supporting the hypothesis of systemic mediators as being the triggers of mucosal damage. In this line, IL-33 plasma levels were elevated in CD, sometimes even after a gluten-free diet (GFD) [7,109,113,114,115]. Strict adjustment to GFD is not always achieved, and systemic levels of certain cytokines such as IL-2 and IL-8 are elevated quickly and dramatically after exposure to gluten [116]. A prolonged systemic inflammation may contribute to long-term complications in untreated CD [117].

In the lamina propria, we observed increased Treg counts, even after avoidance of gluten. Moreover, Treg numbers correlated with oral epithelial damage, as of a decreased e-cadherin expression, and with amphiregulin mRNA levels from Peripheral Blood Mononuclear Cells (PBMCs). This was observed in all experimental groups, suggesting that Tregs may be recruited to repair epithelial damage, therefore displaying a “repair” phenotype [118]. Based on this evidence and given the tolerogenic nature of the oral mucosa, we postulate that oral mucosa remodeling could be a beneficial process to gain access to the underlying immune system. Therefore, CD therapy could benefit from a disrupted epithelium and Treg recruitment for the development of CD immunotherapy (IT), as allergy benefits from sublingual IT (SLIT) [119].

### 4.4. Oral Manifestations of Gastrointestinal Disease

Oral lesions may be a sign of inflammatory bowel disease (IBD). They can appear before the clinically apparent onset of the abdominal disease (5–10% of affected patients), be present during the disease process, or persistent even after the abdominal disease is resolved [120,121,122]. The prevalence rate of oral lesions in IBD is estimated between 20% and 50% in most publications [121,123,124]. Recurrent deep granulomatous aphthous-like ulcers are the most common oral manifestation of Crohn’s Disease [120,125]. In ulcerative colitis (UC), oral lesions are less common; however, pyostomatitis vegetans occurs more commonly in UC than in Crohn’s Disease [121,125].

Bidirectional causality between IBD and oral lesions has also been speculated, specially involving microbiota [126,127]. Dysbiosis in the oral mucosa microbiota has been described in patients with IBD [128,129], while pathogenesis in the oral cavity cause shifts in both oral and intestinal microbiota [126,130]. In this regard, oral microbes may migrate to the gut and exacerbate various gastrointestinal diseases [130].

Macroscopic alterations and histopathological lesions in the oral mucosa have been thoroughly described in many systemic diseases [131,132,133]. However, damage caused by systemic diseases in the stability and integrity of general oral mucosa remains largely unexplored. Molecular and cellular manifestations in the histology of general oral mucosa could be helpful for the diagnosis of oral manifestations of systemic diseases.

### 4.5. Oral Manifestations in Autoimmunity

Many autoimmune dermatoses have a direct effect on the oral mucosa [131,134]. Briefly, autoimmune skin diseases in the oral cavity affect cell–cell adhesion causing intra-epithelial blistering, autoantibodies, or infiltration lymphocytes cause a loss of cell–matrix adhesion or interface inflammation [131]. Dermatoses traditionally linked with oral manifestations include pemphigus, lichen planus, pemphigoid, and geographic tongue, among others [131,132].

Skin damage is also a typical sign of Lupus erythematosus; oral discoid lesions are one of the most prevalent presentations of this disease [135,136,137].

Strong association has been established between periodontal disease (PD) and rheumatoid arthritis (RA) [138], psoriasis [139], or diabetes mellitus [135]; several studies also show evidence of an improved systemic condition after periodontal treatment [140,141,142]. Bidirectional causality between PD and systemic autoimmunity has been speculated [139,143,144,145]. On one hand, the pathogenesis and progression of PD and autoimmune diseases share common cytokines and immunologic factors [139,143,146]; thus, increased levels of these cytokines due to autoimmunity could lead to periodontitis and vice versa. On the other hand, PD could have a triggering effect on autoimmune disease, i.e., select environmental triggers that result in local mucosa inflammation targeting the oral cavity [136,144,147] induce the generation of neoantigens and, among genetically susceptible subjects, the subsequent production of local autoantibodies [147]. Among environmental triggers, a role in pathogenesis has been speculated for bacteria [143,147,148]. In fact, *Porphyromonas gingivalis*, a bacterium associated with PD, has been shown to promote Th17 responses [149].

### 4.6. Oral Mucosa and Systemic Disease

Associations between systemic disease and oral manifestations have been profoundly investigated [135,136,150,151]. Interest in the link between oral and general health manifests not only in epidemiological studies, but also in the potential of the oral mucosa and saliva for diagnosis of systemic diseases [152,153]. A wide variety of systemic diseases have a direct effect in the oral mucosa, including viral infections, dermatoses, hematologic disorders, endocrine diseases, gastrointestinal disorders, malignancy, and autoimmune diseases, among others [123,131,136,154,155,156]. Ulceration, white patches, swellings, pigmentation changes, or periodontal inflammation are the main lesions found in the oral mucosa of systemic disease patients [135,139,143,154]. Although an association is obvious, whether alterations in the oral mucosa are solely a clinical manifestation of the systemic disease or whether they actively contribute to their development has not been clearly established for many diseases [144,147,156,157]. Since the oral cavity is diagnostically accessible, its alterations can be the first indication of a systemic disease and allow for an early diagnosis [120,136,137].

## 5. Potential of Oral Mucosa Characterization in Diagnosis and Treatment

### 5.1. Research Approaches

The use of cell culture models of the oral mucosa helps characterize its barrier properties and its plausible applications in many fields. A huge variety of cell culture models have been developed to mimic specific parts of the oral mucosal barrier, but no ultimate standard has been established, neither for the oral cavity nor for salivary gland epithelia, since the epithelia of the oral cavity (buccal, gingival, etc.) as well as from the different salivary glands (submandibularis, parotid, and sublingualis) present regional variations [158]. Therefore, primary or immortalized keratinocytes and tumor-derived cell lines of different oral mucosa epithelial regions have been set up as monolayers, multilayers, or 3D cultures [158]. Barrier functionality in vitro can be determined by measuring the transepithelial electrical resistance (TEER) of cell layers grown on transwell membranes or by using marker molecules in paracellular transport assays. High TEER values reflect an intact epithelial barrier, i.e., the resistance of the epithelial cell layer to allow ions migrating across it is high. A decrease in TEER values is related to a leakier epithelial layer, linked to a changed expression and/or location of junctional proteins. TEER measurements are influenced by several parameters such as temperature, transwell size, membrane pore-size, and the cell growing medium. More information on TEER measurement techniques can be found in a recent review by Srinivasan et al. [159]. With regard to the paracellular transport assays, fluorescently or radiolabeled molecules are used to assess the integrity of the epithelial barrier [158]. Moreover, histological analyses can be used for the visualization of epithelial structure and junctional protein location by using transmission electron microscopy (TEM) or immunofluorescence microscopy. This imaging techniques support the functional assays [158].

However, the functionality of the oral mucosal barrier is regulated by its microenvironment and altered during diseases. Therefore, future experimental setups and model validations will need to take into account the microenvironment and its influence on barrier properties [160]. One major contributor to the oral cavity microenvironment is saliva, which contains enzymes and mucins, among other molecules. These proteins form an acellular barrier that should be included in in vitro oral cell cultures [161,162]. An advanced in vitro model including mucus has been developed [163]. However, when comparing this model to normal oral keratinocytes, differences in histology as well as in paracellular transport were found [164]. This highlights the complexity of the oral mucosa, which cannot be mimicked by single cell cultures in vitro. Therefore, 3D models including stratified epithelial cell layers, fibroblasts, and endothelial cells have been developed and reviewed elsewhere [165,166,167].

### 5.2. Diagnosis

A systematic review has recently evaluated the ability of mucosal biomarkers to identifying food allergy patients [168]. Twenty-two studies met the eligibility criteria to be included in the systematic review; of those, only two studies analyzed saliva samples focusing on metabolites and sIgA determinations as biomarkers for peanut and milk/egg allergy, respectively [169,170]. However, Tomicic et al. [170] could not find differences in salivary sIgA levels between 4-year-old children who were tolerant and not tolerant to egg and milk consumption. Regarding the study by Peeters et al. [169], the authors describe differences in bulk salivary metabolites before and after peanut ingestion in both peanut-allergic and peanut-tolerant subjects. Unfortunately, they did not identify any of these altered metabolites. Therefore, there is still a lack of salivary biomarkers with a good diagnostic value for food allergy. Until now, gut/fecal samples were preferred to saliva. However, food allergies are systemic disorders, since they are associated with a dysregulation of the mucosal immune system (e.g., breaking of oral tolerance). Therefore, theoretically the identification of biomarkers could be performed on salivary samples since saliva testing is a safe, noninvasive, and low-cost method for diagnosis [171]. Some promising biomarker candidates would include salivary cytokines, such as IL-2 and IL-6, found to be significantly increased in Sjogren’s syndrome, an autoimmune disease [172].

Another study has reviewed salivary protein profiles in the search for biomarkers for oral diseases, such as untreated dental caries, severe periodontal disease, and edentulism [173]. Saliva represents a window of opportunity for modern medicine [174], since several sensitive analytical techniques such as mass spectrometry (MS), reverse transcription polymerase chain reaction (RT-PCR), microarrays, or magnetic resonance spectroscopy (MRS), among others, allow the detection and quantification of many biomarkers. In the last decades, saliva-related research has grown, and new relevant concepts such as point-of-care (POC) diagnostics have emerged [175]. Currently, five “omics” of saliva biomarkers are known (proteome, transcriptome, microRNA (miRNA), metabolome, and microbiome) [176]. In the field of salivanomics, a new landmark has been the discovery of extracellular vesicles. These vesicles contain genetic material and proteins, and play a pivotal role in immune system modulation and inflammation [177]. Saliva is a great source of useful biomarkers in the diagnosis and prognosis of diseases.

There is much less information regarding the features of the oral mucosal barrier as a diagnostic tool. However, the scarce data are promising. In this regard, Rosace et al. [5] suggested a novel role of the oral mucosal barrier in the progression of respiratory to food-induced allergic reactions in patients with profilin-mediated food allergy. In this case, oral epithelial remodeling could be key in the acquisition of a severe allergic phenotype. The accessibility of oral mucosa sampling could facilitate diagnosis and help us to understand disease progression in complex allergic syndromes, such as pathogenesis-related (PR) proteins group 10 or lipid transfer protein (LTP)-mediated food allergy. This will pave the way for the development of new food allergy preventive strategies.

Similarly, oral mucosa biopsy sampling could be helpful in the diagnosis of gastrointestinal diseases, such as CD. Sanchez-Solares et al. [7] described that the oral mucosal barrier of CD patients is compromised, even when they adhere to a GFD. Accordingly, damage in the oral mucosa of GFD-treated patients has been previously reported [178,179]. The oral mucosa remodeling observed in treated CD patients has been suggested not to result from poor adherence to a GFD, but rather as a result of chronic immune stimulation, which induces a delayed immune response with memory T cell generation [179]. In some cases, CD presents with extraintestinal manifestations in the oral cavity such as aphthous ulcers, even after avoiding gluten. Based on these observations, an impaired epithelial barrier could account for these manifestations [180]. Therefore, the characterization of the oral mucosal barrier may be crucial for advancing novel oral biomarkers and immune-based treatments for CD.

Non-celiac gluten sensitivity (NCGS) has recently been identified as a new clinical entity, different from CD, whose prevalence could be higher [181,182,183]. NCGS lacks specific diagnostic markers. HLA-DQ2 and/or HLA-DQ8 genes are present in only 50% of the NCGS patients, and histologic analysis usually does not show specific alterations for CD [184]. These patients may complain of CD-like symptoms, including recurrent oral ulceration receding after a GFD [185]. Recent studies have introduced a new concept according to which adverse effects of dietary antigen exposure may be defined as allergic contact mucositis (ACM) based on a specific oral mucosa patch test (OMPT) [186,187,188]. Picarelli et al. [189] evaluated local and general reactions triggered by direct contact with gluten. The gluten OMPT (GOMPT) showed positive results in 75% of the NCGS patients and in none of the healthy controls, proving to be a promising tool for NCGS diagnosis. However, GOMPT also showed positive results in 25% of the treated CD patients. It remains unclear whether there are common immunopathogenic mechanisms among gluten-dependent disorders.

### 5.3. Treatment

#### 5.3.1. Micronutrients

Several micronutrients and nutraceuticals have been proven capable of inducing structural changes in epithelial TJ complexes that result in reduced leakiness across many different epithelial barriers in vitro or in experimental models [190,191,192,193,194,195,196,197,198,199]. These compounds range from dietary constituents such as the procyanidins of grape seeds to metabolic end-products of the gastrointestinal microbiome such as butyrate [200,201]. Rybakovsky et al. [202] found that zinc, quercetin, retinoic acid (RA), and the boswellic acid derivative, AKBA (3-O-acetyl-11-keto-β-boswellic acid) were also able to induce compositional changes in oral epithelial TJs. Retinoic acid was found to enhance the expression of the TJ proteins claudin-4 and occludin in the gingiva [203]. Based on these studies, micronutrient-based oral prophylaxis would improve oral epithelial barrier function basally and/or reduce the induced leakiness caused by different diseases. Whether the exposure to these micronutrients via the diet would be sufficient to induce substantial changes in the oral epithelial barrier or whether their supplementation in higher amounts is required is a matter of future research.

In regard to food allergies, a study found baicalein (5,6,7-trihydroxyflavone), a flavonoid from *Scutellaria baicalensis* that is used in oriental herbal medicine, to enhance intestinal barrier function through the regulation of TJ in a mouse model of food allergy. Moreover, baicalein treatment induced the differentiation of Tregs via aryl hydrocarbon receptors (AhRs). Therefore, baicalein could be used as an effective immune regulator for the treatment of food allergies [204]. Another study investigated the use of Galectin-1 in a mouse model of peanut allergy. In this model, mice were sensitized to peanut extracts (PE) via the buccal mucosa with or without Galectin-1. The authors found that the pathogenesis of the oral-intestinal allergy syndrome (OIAS) was inhibited by Galectin-1 by suppressing micro RNA (miRNA)-98 and reversing the expression of IL-10 in CD14+ cells in the intestine [205].

In this regard, the study by Antunes et al. [206] found that oral supplementation with capsaicin reduced macrophage infiltration and IL-33 production in proximal jejunum in a mouse model of OVA allergy. The study by Yoshimoto et al. [207] explored the effects of the pathohistological changes in oral epithelia on pain by using labial mucosa samples from patients with suspected Sjogren’s syndrome (SS) because they frequently experience pain and discomfort. The disrupted epithelia contained high numbers of infiltrating macrophages and remodeled cell adhesion molecules such as filamentous actin, E-cadherin, or β-catenin. These findings would be very useful for treating recurrent or chronic oral pain, since according to the authors macrophages may have an active role in degenerative processes with either pathological or reparative properties. Therefore, a reduction in macrophage infiltration may help alleviate pain. In any case, further studies are needed to characterize the role of epithelial macrophages.

#### 5.3.2. Oral Immunotherapy or Desensitization

There is broad experience in using oral mucosa as a drug delivery site. From viral attenuated vaccines [208] to small molecules, the oral mucosa possesses unique features that confer significant advantages over the gastric route. Drugs in general are absorbed faster and access directly the blood circulation bypassing the liver; therefore, avoiding hepatic clearance [209]. The convenience of oral formulation has led to significant efforts in the formulation of oral vaccines [210,211,212,213]; however, oral mucosa delivery is not generally considered for conventional vaccines due to a limited accessibility and its low immunogenic potential.

A completely different approach was led in the last decades of the 20th century to exploratory clinical trials of the sublingual route for allergen immunotherapy. Among the tissues and organs initially exposed to allergens such as the nose, the lungs, and the skin, the allergic inflammatory reaction spreads to distant sites, which finally results in a systemic disease. By contrast, the oral mucosa is a tolerogenic site regarding the immunologic response to allergens. The oral mucosa is characterized by an abundance of DCs and Tregs cells. Moreover, the presence of effector inflammatory cells, namely, mast cells and eosinophils, in the oral mucosa is negligible. This accounts for the good safety of the sublingual administration of allergen immunotherapy [214,215]. The increasing evidence of efficacy and a better safety profile [216] led for the first time to extensive product development programs for sublingual vaccines that constitute the first allergen immunotherapy products registered worldwide. Among them, GRAZAX possesses a whole series of short, medium, and long-term clinical trials that allow the understanding of the underlying immunological mechanisms and their link to clinical effects [217]. In contrast to conventional vaccines, allergen immunotherapy (AIT) intends to regulate an already established immunological response, mainly by driving effector cell desensitization as well as inducing regulatory responses. In this sense, oral mucosa represents an immune-privileged site due to its tolerogenic nature.

Early clinical benefits of AIT are driven by effector cell desensitization. Simultaneously, a regulatory response is initiated, detected by changes in functionality of dendritic cells [218]. The desensitization drives the clinical benefit during the first two years of AIT. During this phase, regulatory response continues as a progression that is only consolidated in the third year of treatment [119,219,220]. A key question about these mechanisms is their potential interdependence. Mast cells are strong producers of inflammatory mediators; thus, their desensitization in the early treatment phase could be essential for the control of inflammation and the onset of the later regulatory response. This fact could be critical for the potential applicability of this approach in autoimmune diseases, where desensitization is not involved. In the last years, multiple trials in AIT that only focused on T regulation have failed so far, but all of them were carried out for less than two years, a period that is not enough to consolidate T cell regulation [220].

Exploratory trials on SLIT have been carried out mainly with two food allergy models. The first ones were focused on LTP allergy [221,222,223,224]. Accumulated evidence supports a clear clinical benefit. Approximately 90% of patients suffering from anaphylactic reactions induced by peach tolerated a full piece of fruit (3 mg of allergen) after the administration of 10 µg of Pru p 3 for one year. Similarly, Fleischer et al. [225] reported a significant effect of SLIT in peanut allergy. After a three-year follow-up [226], the effect was maintained, but it was moderate. More studies would be needed to understand and evaluate the potential role of AIT in food allergy. With the available data, we can conclude that the threshold for suffering severe reactions might increase, with an excellent tolerability. This treatment would at least minimize the risk of suffering life threatening reactions upon unadvertised exposure.

There is also a need to understand the interaction of the antigen with the immunological system during SLIT. Since the oral cavity possesses specialized immunological organs [227], such as the lingual tonsil that is a part of the Waldeyer ring, this could be relevant for the induction of a regulatory response [228,229]. Several studies have demonstrated that topical application of hapten onto buccal mucosa induces both the migration of DCs and hapten-specific T-cell responses. Oral lymphoid foci may act as sites in which antigen-experienced mature B cells interact with T cells to promote the expansion of Treg cells in tolerance induced by allergen application via the oral mucosa. In fact, SLIT leads to tolerance against allergens possibly via the redirection of allergen-specific Th2 cells to Th1 cells and the generation of peripheral Treg cells [41]. On the contrary, children with peanut or egg allergy showed a decrease in Treg cell percentage after allergen exposure [230]. As discussed by Satitsuksanoa et al. [230], oral immunotherapy, the only known therapy for food allergies, increases Treg cell function, hypomethylation of the FOXP3 gene, and the number of FOXP3-positive cells.

As previously described, the oral mucosa undergoes progressive structural changes and increasing inflammatory infiltrates in food allergy associated with respiratory allergic progression [5] or in severe respiratory allergic patients but in the absence of food allergy [6]. Moreover, a strong increase in both sIgE and IL4-secreting cells has been described in the first month of AIT [219]. Therefore, the oral mucosa barrier impairment associated with allergic inflammation might be critical for the SLIT effect [6,202]. In particular for effector cell desensitization, which has been proven to be dose dependent, this barrier impairment and associated access to effector cells might be required. In fact, analyzing the AIT effect over a series of almost 2000 patients treated with grass, SLIT [231] patients with low sIgE levels (lower tercile) to grass’ major allergens did not show a clinical benefit in the first year of treatment, suggesting that effector cell desensitization did not happen.

Recent accumulating evidence suggests that a possible “mouth–gut axis” may exist in the pathogenesis of gastrointestinal diseases [130], and as such, oral mucosa changes associated to inflammatory diseases could serve as model to understand global barrier impairment, that could be in the genesis of food allergic sensitization, CD, etc. We also need to understand how systemic inflammatory factors may affect the integrity of the oral epithelium and to further investigate common metabolic and transcriptomic fingerprints that might be useful for the identification of new therapeutic targets to prevent allergic progression [6].

The oral mucosa is highly enriched in CD4+ T cells, which contrasts to the intestinal mucosa that contain CD8αα+ T cells and CD103+CD69+ tissue-resident memory CD8+ T cells [232]. This is important for the development of tolerization strategies in CD. In the study by Sanchez-Solares et al. [7], no significant differences were found for CD4+, CD8+, or CD3+ cell populations between CD patients and healthy subjects. However, Bardellini et al. [178] described increased CD3+ numbers in the stromal papillae that were reduced in GFD. Nonetheless, we observed that the relative numbers of epithelial lymphocytes diminish after gluten avoidance. Moreover, the vast majority of the oral intraepithelial lymphocytes (IELs) lacked γδ-T cell receptor expression. This was also observed by Lähteenoja et al. [179], who suggested that NK cells were mainly responsible for lymphocyte recruitment; thus, NK cells may also be involved in the remodeling process of the oral mucosa. Conversely, γδ+ cells were found at the gingiva in a mouse model of periodontitis [233]. In addition, they produced amphiregulin, which guaranteed oral mucosa homeostasis. In our study, Treg population was greatly increased in the oral mucosa of CD patients [7]. Moreover, Treg abundance was inversely correlated with E-cadherin expression and directly correlated with peripheral amphiregulin expression. Therefore, we postulate that Tregs were recruited to prevent further epithelial damage and maintain barrier integrity, as reported previously [6,234]. However, it remains to be determined whether these Tregs have an impaired suppressive function, as previously described [235,236]. In this sense, amphiregulin has been previously reported to display repair/remodeling features in acute graft-versus-host disease (aGVHD) [237] or infant viral bronchiolitis [238] constraining chronic inflammation [239]. Therefore, amphiregulin and Treg analyses in the oral mucosa could be of potential use as targets for oral tolerization approaches.

## 6. Conclusions

The oral mucosa is a immunocompetent site that is constantly exposed to foods; therefore, its primary role is tolerogenic. However, the disruption of the integrity of the oral mucosa due to inflamation induces further inflammation and eventually immune systemic responses. The characterization of its histological structure and immunological features makes it possible to assess its role in the onset, progression, and final outcome of a variety of inflammatory diseases, as has been reviewed here. Studying the oral mucosa is not only helpful to depict immune diseases, but it is also essential to understand treatment efficacy, as for SLIT in allergic diseases, and to develop novel diagnostic and therapeutic strategies.

Despite the increasing knowledge recently generated regarding the oral mucosa, there is still a significant lack of description of the mechanisms underlying the local immune response taking place in the oral mucosa. Moreover, it is critical to understand how local mucosa-associated immune responses are connected to systemic immune outcomes. Therefore, deeper analyses of these processes may improve the management and development of more accurate and less invasive diagnostic strategies as well as potentiate the advance of novel therapeutic approaches targeting the oral mucosa.

## Data Availability

Data sharing not applicable.
